# Quality Dynamics of Beef Bottom Round During 2-Month Frozen Storage (−18 °C) and Week-Long Refrigeration (4 °C)

**DOI:** 10.3390/metabo15050294

**Published:** 2025-04-29

**Authors:** Yue Song, Wenbo Hou, Mengliu Zhu, Otobong Donald Akan, Yanxia Xing, Yang Yu, Bo Li, He Zhu

**Affiliations:** 1 College of Food Science and Engineering, Shandong Agriculture and Engineering University, Jinan 250100, China; z2023120@sdaeu.edu.cn (Y.S.); z2023124@sdaeu.edu.cn (W.H.); z2021014@sdaeu.edu.cn (M.Z.); z2014007@sdaeu.edu.cn (Y.X.); z2020030@sdaeu.edu.cn (B.L.); 2 Microbiology Department, Akwa Ibom State University, Uyo 999062, Nigeria; otobongakan@aksu.edu.ng

**Keywords:** beef bottom round, storage, physicochemical properties, volatile organic compounds

## Abstract

**Background/Objectives**: The present study aimed to investigate the effects of frozen (−18 °C) and refrigerated (4 °C) storage conditions on several beef bottom round quality parameters. **Methods**: Fresh beef bottom round samples were stored under both frozen (−18 °C) and refrigerated (4 °C) conditions. For frozen samples, the pH, color, cooking loss, thawing loss, centrifugal loss, drip loss, moisture content, shear force, TBARS and TPA were measured at 0, 30 and 60 days. For refrigerated samples, the pH and color were analyzed at 0, 1, 3, 5 and 7 days, and the differential metabolites were also identified based on the VOCs analysis combined with multivariate statistical analysis. **Results**: The surface color (L*, a*, and b*) of the beef bottom round became darker during both the frozen and refrigerated periods of prolonged storage. The samples’ pH significantly declined (*p* < 0.05) during the frozen storage but alternated (initially reduced and then increased) under refrigerated conditions. Additionally, the frozen treatment led to a significant change (*p* < 0.05) in the texture profile. The thiobarbituric acid reactive substance (TBAR) values, shear force, cooking loss, thawing loss, centrifugal loss, and drip loss increased significantly with an extended frozen storage time, while the moisture content was significantly lower (*p* < 0.05). Moreover, nine volatile organic compounds (VOCs) were identified as potential determinants of beef bottom quality during refrigerated storage. **Conclusions**: The findings can contribute to a deeper understanding of quality variations during frozen storage and refrigerated storage, and provide new thoughts to improve preservation and storage strategies for the beef bottom round.

## 1. Introduction

Beef is highly nutritive due to its high protein and rich Fe, Zn, and vitamin contents. Driven by changes, including those in human population, income, and consumer preferences, the average of demand for protein from beef has rapidly increased in recent decades, especially in Asia and Africa [1]. Recent statistics show that the consumption of beef protein peaked in 2024, consumed mostly in first- and second-tier cities [2]. This applies especially to the beef bottom round, which is much higher in protein content. Existing studies show that the content of protein in the bottom round is much higher than in other cuts. Soohyun Cho reported that the content of protein was up to 22.12%, which was only exceeded by the eye of round cut, but is much higher than that in tenderloin, loin, striploin, chuck roll, chuck tender, oyster blade, short plate, top plate, top round, and brisket of cattle [3]. And, the researcher Ji-Hey Jeon reported that the total content of amino acids in beef bottom round were much higher than that in tenderloin, sirloin, brisket, and flank. Amino acids serve as the basic structural units of proteins, linked by peptide bonds to form polypeptide chains [4]. The elevated protein concentration is intrinsically linked to the anatomical particularities of this muscle group. The bottom round belongs to locomotor muscles, which are rich in type I slow-twitch muscle fibers. Type I slow-twitch oxidative fibers demonstrate significant protein accumulation traits, with this biochemical property being most pronounced in weight-bearing musculature including the semimembranosus and biceps femoris of the beef round [5]. In addition, the fat content of the beef bottom round is moderate, and the majority of it consists of unsaturated fatty acids, which are beneficial for humans [6].

Freezing (−18 °C) and refrigeration (4 °C) are commonly used preservation methods for meat products. Frozen storage under −18 °C may effectively inhibit certain enzymatic and microbial activities, so it is most widely used in the meat industry [7]. And, refrigerated storage may reduce the reproduction rate of microorganisms in the short term; it is more useful for family use. Although low-temperature treatment can extend the shelf life of meat, studies have found that the properties of frozen meat are negatively affected over prolonged storage periods [8].

As living standard rise, people adopt higher requirements for beef quality and freshness. Nowadays, numerous studies have already been conducted focused on this topic. For instance, Mingming Zhu reported that, after 7 days of storage at 4 °C, the meat exhibited color deterioration and its water-holding capacity was significantly reduced under frozen conditions (−18 °C) [9]. And, Renata Stanislawczyk found that frozen storage could influence the quality of horse meat, especially in terms of the water retaining capacity and the pH [10]. In addition to the physicochemical properties, VOC analysis is also an effective method to evaluate the quality dynamics of meat. Sam Al-Dalali investigated the untargeted metabolites of roasted beef meat; the results show that frozen storage affected the differential metabolites [11]. Above all, recent studies have mainly focused on beef, horse, chicken, or other livestock meat, but on less specific parts of the livestock. In particular, few studies have focused on the beef bottom round. Thus, it is necessary to investigate the quality dynamics of the beef bottom round during frozen and refrigerated storage. The aim of our study was to determine the beef’s physicochemical properties, including color, pH, WHC, TBARS, shear force, and TPA. Also, the VOC analysis was conducted based on the GC-MSD, to conduct a comprehensive analysis of the effects of frozen and refrigerated storage on the beef bottom round, and provide scientific guidelines for families and the meat industry.

## 2. Materials and Methods

### 2.1. Chemicals Used

The potassium chloride (KCl) was purchased from the Damao Chemical Reagent Factory (Tianjin, China). The trichloroacetic acid (TCA), absolute ethyl alcohol, and thiobarbituric acid were purchased from Sinopharm Chemical Reagent Co., Ltd. (Shanghai, China). The potassium acid phthalate (pH = 4.00) and the mixed phosphate (pH = 6.86) were purchased from INASE Scientific Instrument Co., Ltd. (Shanghai, China). The potassium bromide was purchased from Shandong Keyuan Biochemical Co., Ltd. (Jinan, China).

### 2.2. Beef Samples

Beef bottom round cuts (from the left hip section) were obtained from 6 Chinese Luxi yellow cattle carcasses (about 20 months old and fed the same diet) at a commercial abattoir. The cattle always arrived at the lairage at 5 pm in order to prevent animal stress. All cattle were subjected to a 24 h feed/fasting cycle and 2 h of water withholding before slaughter. Fasting from water must be implemented 4 h before slaughter. Sample collection was performed at exactly 12 h after slaughter. The initial pH and the ultimate pH at the time of deboning were 6.5–6.7 and 6.0–6.2, respectively. The samples were wrapped at 0–4 °C and delivered to the laboratory in 1.5 h. Each beef bottom round was cut into 8 sections (with an average weight of about 200 g/section). Based on analogous fat distribution and homogeneous visual parameters, these sections were randomly assigned to 0, 30, 60 days of frozen storage and 0, 1, 3, 5, and 7 days of refrigerated storage. Five replicates were set for physicochemical analysis, and three replicates for VOC analysis.

### 2.3. Quality Assessment

#### 2.3.1. Color

A colorimeter (3 nh handheld spectrophotometer YS 3010, Guangdong Sunence Intelligent Technology Co., Ltd. Guangzhou, China) was used for color measurement. The beef bottom round samples’ color values L* (lightness), a* (redness), and b* (yellowness) were measured. The colorimeter was calibrated before each measurement.

#### 2.3.2. pH

The pH measurement of the beef bottom round samples was performed using a Mettler digital pH meter (Zurich, Switzerland). The pH meter was calibrated at room temperature using standard solutions with pH values of pH buffer solutions (25 °C, pH = 4.01, 7.00, 9.20, and 10.00). The pH was measured after blending a 5 g beef sample with 50 mL 0.1 M KCl solutions and equilibrating for 10 min [12].

#### 2.3.3. Cooking Loss

Beef bottom round samples measuring 2 cm × 2 cm × 2 cm were sealed in high-temperature cooking bags and placed in a water bath at 85 °C. Each beef bottom round sample was cooked until the internal temperature reached 75 °C. After cooking, the beef bottom round samples were left to cool at room temperature for 30 min. Excess moisture was removed by dapping with paper wipers [13]. Sample weights measured before and after cooking were used to calculate the cooking loss as stated in Equation (1).

Cooking loss (%) = [(initial raw beef weight − weight after cooking)/initial raw beef weight] × 100(1)

#### 2.3.4. Thawing Loss

Exactly 10 g of frozen beef bottom round samples were left to thaw under 4 °C conditions for 24 h. Then, the surface moisture was wiped away with a paper towel [14]. The thawing loss was calculated using Equation (2) below.

Thawing loss (%) = [(frozen beef weight − thawed beef sample)/frozen beef weight] × 100(2)

#### 2.3.5. Centrifugal Loss

Thawed beef bottom round samples were centrifuged at 2500 rpm for 10 min at 4 °C [15]. The centrifugal loss was calculated using Equation (3) below.

Centrifugal loss (%) = [(sample weight before centrifugation − sample weight after centrifugation)/Sample weight before centrifugation] × 100(3)

#### 2.3.6. Drip Loss

Thawed beef bottom round samples were weighed, recorded, and hung at 4 °C for 24 h. Surface moisture was wiped away with a paper towel before weighing [16]. Drip loss was calculated as the percentage of weight loss relative to the initial sample weight before hanging using Equation (4) below.

Drip loss (%) = [(Beef sample weight before hanging − beef sample weight after hanging)/Beef sample wight before hanging] × 100(4)

#### 2.3.7. Moisture Content

Determined according to the “Determination of moisture in foods” (GB 5009.3-2016) published by the National Health and Family Planning Commission of the People’s Republic of China and the Standardization Administration of China [17].

Exactly 3 g of beef bottom round sample was placed in a dried glass weighing bottle, and the initial weight was recorded. Subsequently, beef samples were dried in an oven at 105 °C, and left to attain a constant weight (which was recorded). The moisture content of the beef samples was calculated using Equation (5) below.

Moisture (%) = [(initial beef sample weight − dried beef sample weight)/raw beef sample weight] × 100(5)

#### 2.3.8. Shear Force

The Warner–Bratzler shear force of the beef bottom round samples was determined according to the method described by Silva and co-workers with some modifications [18]. Firstly, the beef bottom round samples (3 cm × 3 cm × 2 cm) were cooled at 4 °C for 24 h. Then, the shear force test was performed utilizing a TA-XT Plus texture analyzer (Stable Micro Systems, Godalming, UK) equipped with an HDP/BSK probe. The texture analyzer was calibrated for a pre-test speed of 2 mm/s, a test speed of 2 mm/s, and a post-test speed of 1 mm/s, with the device programmed to move 20 mm at the end of the three phases. The shear force (kg) was calculated as the maximum positive value of the curve.

#### 2.3.9. Thiobarbituric Acid-Reactive Substances (TBARSs)

The method by Piranavatharsan and co-workers was followed to determine the TBARS of the beef bottom round samples [19]. Firstly, 2 g of beef bottom round samples were homogenized in 20 mL of 10% TCA solutions using a vortex for 5 min and centrifuged at 12,000 rpm for 10 min. Secondly, 5 mL supernatant was added to a test tube containing 5 mL 0.02 M thiobarbituric acid (TBA, 3% in ethanol) and vortexed. The solution was heated in a water bath at 100 °C for 20 min. After cooling to room temperature, the sample was centrifuged at 12,000 rpm. The absorbance of the supernatant was measured at 532 nm. The TBARS values were expressed as mg malondialdehyde (MDA)/kg sample.

#### 2.3.10. Texture Profile Analysis (TPA)

The TPA of the samples was carried out using a TA-XT plus Texture Analyzer (Stable Micro Systems, UK) equipped with a P/36R probe to measure hardness, cohesiveness, springiness, gumminess, chewiness, and resilience. The TPA was performed with the frozen beef bottom round samples (on the first, 30th, and 60th day). Firstly, the beef samples (2 cm × 2 cm × 2 cm) were cooled at 4 °C for 24 h. The samples were subjected to a two-cycle compression test. The test conditions were as follows: 1 mm/s pre-test and post-test speeds and a 2 mm/s test speed.

### 2.4. GC-MS Analysis

GC-MS analysis of the beef bottom round samples was performed using an Agilent 7890A GC coupled with an Agilent 5975C MSD quadruple detector with an electron ionization (EI) source (Agilent Technologies, Santa Clara, CA, USA). The separation of target metabolites was performed on HP-5MS (30 m × 250 µm i.d., 0.25 µm film thickness), and helium was selected as the carrier gas at a flow rate of 1 mL/min. The temperature of the interface was set at 250 °C. The initial temperature of the oven was held at 35 °C for 2 min, increasing to 130 °C at 5 °C/min, and held for 2 min. Then, the temperature was increased to 230 °C at 8 °C/min and held at this temperature for 5 min.

The MS was performed in electron ionization mode using electron energy of 70 eV at 250 °C, the scan range was *m*/*z* 30–400, and the solvent delay was 1 min.

### 2.5. Statistical Analysis

In this study, average data from five replications were presented as mean values with SE. Statistical analysis was performed using SPSS statistics software (version 22.0; IBM, Armonk, NY, USA). The bar chart was drawn by the GraphPad Prism software (version 10.0; San Diego, CA, USA). The raw data acquired from GC-MSD were pre-processed by MSD Chemstation software (version E. 02.02.1431; Agilent Technologies, USA), and then the pre-processed data were imported into SIMCA software (version 14.1; Umetrics, Sweden) to perform the principal component analysis (PCA) and partial least squares discrimination analysis (PLS-DA). The PCA and PLS-DA were employed to analyze the differences among five groups and to identify the chemical markers. The statistical significance was set at *p* < 0.05.

## 3. Results and Discussion

### 3.1. Quality Parameters of Beef During Long-Term Frozen Storage

#### 3.1.1. Color and pH

**Figure 1:**
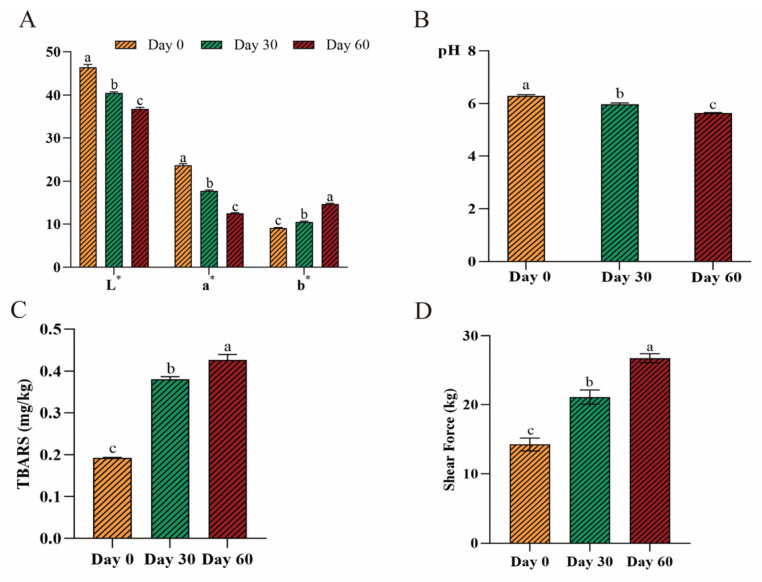
Changes in the quality of beef bottom round due to frozen temperature: (**A**) change in color, (**B**) change in pH, (**C**) change in shear force, and (**D**) change in TBARSs (*n* = 5). Bars within the same storage time without the same letter (a, b, and c) were significantly different according to the Duncan post hoc test (*p* < 0.05).

The surface color of the beef bottom rounds was significantly affected by extended storage time. As shown in Figure 1A, compared with fresh beef bottom round samples (day 0), the surface (L* value) color quality showed a 12.88 percentage point decrease and a 20.74 percentage point decrease on day 30 and day 60. In Wenxin Wang’s study, they also found that all beef steak sample groups exhibited a significant decrease in the L* and a* values [20]. The L* value represents the depth of black and white, which ranges from 0 to 100 (and is a direct measurement of light reflection on the beef samples’ surface). It was closely relevant to the water content and mobility of water molecules, hemoglobin, and cytochrome c; all these factors together resulted in the changes in the raw meat color [21]. In our study, the water content decreased with the prolonged storage time, so the accumulation of cytochrome led to a darker beef bottom round surface color. Also, protein denaturation and the generation of exudate could have affected the L* value [22].

The redness of the beef bottom round sample surfaces (the a* value) was significantly decreased with increased frozen (−18 °C) storage duration. As shown in Figure 1A, the a* and the L* values followed the same trend. Compared with the fresh beef bottom round samples (day 0), the surface (a* value) color quality showed a 25.02 percentage point decrease and a 47.11 percentage point decrease on day 30 and day 60. The color mainly depends on the concentration and the status of Metmyoglobin, and the low temperature inhibited the activity of Metmyoglobin reductase, which led to a huge accumulation of Metmyoglobin and this resulted in decreased a* value and beef surface color [23]. Also, the sensitivity of myoglobin oxidation increased; this was also a factor that led to the darkening of the beef bottom round surface color. Moreover, juice leakage would increase with a prolonged storage time and some water-soluble pigments would be lost simultaneously, which could result in the serious destruction of myoglobin [24].

Along with color characteristics, the b* value is also important as it measures the yellowness of the beef bottom round surfaces. As shown in Figure 1A, the b* value showed a 15.71 percentage point increase and a 61.44 percentage point increase on day 30 and day 60, compared with the fresh beef bottom round samples (day 0). The b* value on day 60 was the highest and was significantly different from those recorded for day 0 and day 30 (*p* < 0.05). This result was consistent with the study by Gabriala M. Bernardez-Morales, which reported that even under the protection of packaging film, the b* value still increases with prolonged cold storage at about −20 °C [25]. This is because muscle exposure to oxygen leads to lipid oxidation. Free radicals generated from the lipid oxidation process destroy pigments in muscles, which affects the color-change process [26].

pH is an important factor for evaluating meat quality. The pH of the beef bottom round samples exhibited a 5.24 decrease on day 30 and a 10.39 decrease on day 60 during the frozen storage period, compared to day 0, as shown in Figure 1B. This is because during the frozen storage period, the concentration and composition of salt could change the pH and then impact the reactivity of proteins and enzyme activity [27]. Due to the frozen treatment, a large quantity of H^+^ is released during the protein denaturation process throughout the storage period; this is the major cause of the pH decline. Moreover, glycogenolysis, the loss of water and soluble matter, and the production of acidoids are caused by the freezing process [28,29]. The pH on day 30 was significantly lower than on day 0; this was because of the aging of the beef. During this process, the muscle glycogen and ATP could produce lactic acid, phosphoric acid, and creatine, which led to the decline of pH [30].

#### 3.1.2. TBARS

The evaluation of TBARSs is generally regarded as the indicator for the occurrence of lipid oxidation in meat during a frozen storage period [31]. Figure 1C shows that the TBARS exhibited a 98.06 percentage point increase on day 30 and a 122.47 percentage point increase on day 60, which is consistent with the study by Cheng Wu and his team [32]. This is because MDA, as the main reaction product in lipid oxidation, could accumulate with a prolonged storage time, and the MDA could react with TBA [33]. The results illustrate that lipid hydrolysis and oxidation also occur under frozen conditions; the low temperatures cannot completely prevent beef spoilage as the metal ions and reaction oxygen can both promote lipid oxidation.

#### 3.1.3. Shear Force

As shown in Figure 1D, the shear force of the beef samples showed a 48.09 percentage point increase and an 87.66 percentage point increase on day 30 and day 60, compared to that recorded for the fresh (day 0) beef sample. The study indicates that the shear force and water-holding capacity of the beef samples correlate positively [34]. In our study, the moisture content decreased significantly during the prolonged frozen storage, which can verify this statement. And, our results were also consistent with the findings of Lagessted and co-workers [35]. In addition, the researcher Benjamin W.B Holmam also reported that the shear force of beef increased with a prolonged frozen storage time over 1 year [36]. And, Xu Zequan and his team also found that the shear force of pork significant increased with a prolonged frozen storage time at −18 °C [37].

The explanation is the formation of large crystals with extended storage time; these crystals puncture the cell membrane, resulting in fluid leakage and the lessening of fluids that could hydrate the beef muscle fibers. So, more fibers per surface area undergo a decrease in tenderness [38]. Moreover, with the extension of frozen storage, protein denaturation is followed by degradation, muscle fibers are damaged, and the integrity of the muscle is compromised, and this promotes oxidation or even the freeze-based denaturation of the meat, leading to an increase in hardness [39].

#### 3.1.4. Cooking Loss, Thawing Loss, Centrifugal Loss, Drip Loss, and Moisture Content

**Figure 2:**
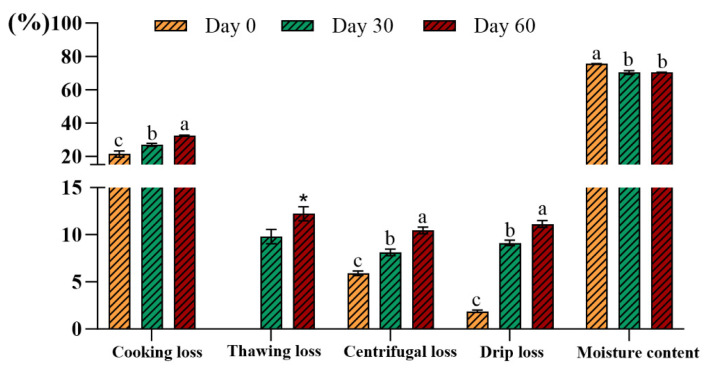
Changes in the water-holding capacity of beef bottom rounds stored at −18 °C during storage duration (n = 5). The * *p*-value < 0.05 was considered statistically significant. Bars within the same quality parameter without the same letter (a, b, and c) were significantly according to the Duncan post hoc test (*p* < 0.05).

The ability of beef bottom round samples to retain their inherent water (WHC) even when water is added during processing is a measure of their quality. As shown in Figure 2, compared to the day 0 sample, the cooking loss showed a 26.22 percentage point increase and a 32.43 percentage point increase on day 30 and day 60. And, the thawing loss exhibited a 24.73 percentage point increase on day 60, compared to day 30. As for the centrifugal loss, this showed a 37.40 percentage point increase on day 30 and a 76.57 percentage point increase on day 60. In addition, compared to day 0, the drip loss increased by about 4-fold and about 5-fold on day 30 and day 60, respectively. Remarkably, the moisture content of the beef bottom round samples showed a 7.0 percentage point decrease after 30 days, but no significant difference between the moisture contents of day 30 and day 60. The results in our study agreed with the findings of Yuqian Xu [40]. The decline in the beef bottom round samples’ water-holding capacity may be attributed to several reasons: First, the oxidation of sulfhydryl groups or disulfides form disulfide bonds, which causes protein aggregation and thus reduces the solubility of proteins [41]. Second, the increase in intracellular ionic strength causes protein denaturation, causing myofibrillar proteins to lose their water retention and thus release exudate or free water [42].

#### 3.1.5. TPA

Table 1 shows that the hardness, springiness, cohesiveness, chewiness, and resilience of the beef bottom round samples changed with storage (−18 °C) time. The hardness of the beef bottom round samples declined by 50.30 percentage points on day 30 and 77.24 percentage points on day 60 during the storage period. The harder the beef bottom round, the higher the chewing efforts required; therefore, with decreased hardness, the chewiness of the beef also decreased. Compared to day 0, the chewiness of the beef bottom round samples showed a4 7.71 percentage point decrease on day 30 and a 61.71 percentage point decrease on day 60. The researcher Mi Hye Park also found that the chewiness of beef was significant decreased by refrigeration [43]. The adhesiveness of the beef bottom round samples declined 31.74 percentage points on day 30 and 72.04 percentage points on day 60, compared to day 0. And, the springiness of the beef bottom round samples decreased 48.84 percentage points and 58.14 percentage points on day 30 and day 60, compared day 0. In addition, the gumminess showed a 47.19 percentage point decrease and a 66.72 percentage point decrease on day 30 and day 60, compared to day 0. The results of both gumminess and springiness are also consistent with Mi Hye Park [43].

Retrospective studies have found that the texture characteristics of beef samples correlate to their structural stability; when frozen beef samples are thawed, ice crystals in the beef tissues puncture the cells, resulting in the collapse of the cell structure, deteriorating the stability and hardness of the beef tissues [44]. With lengthy freezing times, ice crystals grow in the tissue, destabilizing the beef bottom round’s structure. This correlates with the beef bottom round’s reduced hardness at day 60, compared to day 30. Also, the freeze–thaw process causes the loss of cell fluid, which increases the number of muscle fibers per unit volume and increases the cohesion and elasticity of the beef samples. These gradually decrease with decreasing water content in the cells [45]. However, with extensive frozen storage periods, there was no significant change in the resilience of the beef bottom round samples (*p* > 0.05).

### 3.2. Quality Parameters of Beef Samples During Refrigerated Storage

#### 3.2.1. pH and Color

**Figure 3:**
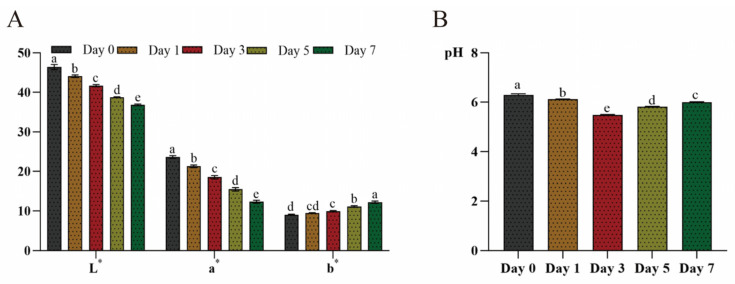
Changes in beef bottom round sample color and pH. (**A**) changes in color quality (L*, a*, and b*) and (**B**) pH in beef during storage (4 °C) (n = 5). Bars within the same quality parameter or same storage time without the same letter (a, b, c, d and e) were significantly different according to the Duncan post hoc test (*p* < 0.05).

This study also checked the impact of refrigeration (4 °C) temperature on the beef bottom round samples’ color and pH over seven days. Figure 3A shows that the L* and a* values significantly declined towards the 7th day of storage at 4 °C (*p* < 0.05); the L* value of the beef’s surface color showed a 20.69 percentage point decrease on day 7, compared to day 0, and the a* value exhibited a 47.75 percentage point decrease on day 7, compared to day 0. The surface color depends on the myoglobin, hemoglobin, and tiny amounts of colored metabolites. However, combined with oxygen, hemoglobin, and myoglobin, it transforms into oxyhemoglobin and oxymyoglobin, which thereafter will change to metmyoglobin, appearing deep red in color [46]. Conversely, the beef samples’ b* values showed a 35.23 percentage point increase on day 7, compared to day 0. This illustrates that the surface color of beef gradually changes to yellow as storage progresses. This discoloration results from lipid oxidation—beef bottom round samples’ exposure to oxygen leads to the oxidation of phospholipids and neutral lipids [47].

The stored beef bottom round samples’ pH changed significantly during the 7-day storage duration (*p* < 0.05), as shown in Figure 3B. The results show that, compared to day 0, the pH decreased 12.79 percentage points on day 3, but only 4.67 percentage points on day 7. Firstly, in slaughtering the meat source, oxygen disrupts beef muscles, and glycogenolysis moves from aerobic to anaerobic metabolism, and the combination of these processes creates acidic conditions [48]. Also, the degradation of ATP releases H^+^ and decreases pH, and with sufficient glycogen, the low pH inhibits enzymatic activity in the anaerobic glycolysis process; with discontinued glycogenolysis the pH starts to increase [49]. The extended storage (4 °C) of beef bottom round samples encourages microorganisms that secrete proteolytic enzymes, further degrading the beef muscle proteins into amino acids and polypeptides. With protein degradation and the release of basic groups, the pH of the beef bottom round samples increases [32]. This underscores pH as a definitive indicator of the freshness or staleness of a meat product.

#### 3.2.2. Unsupervised and Supervised Multigroup Analysis

**Figure 4:**
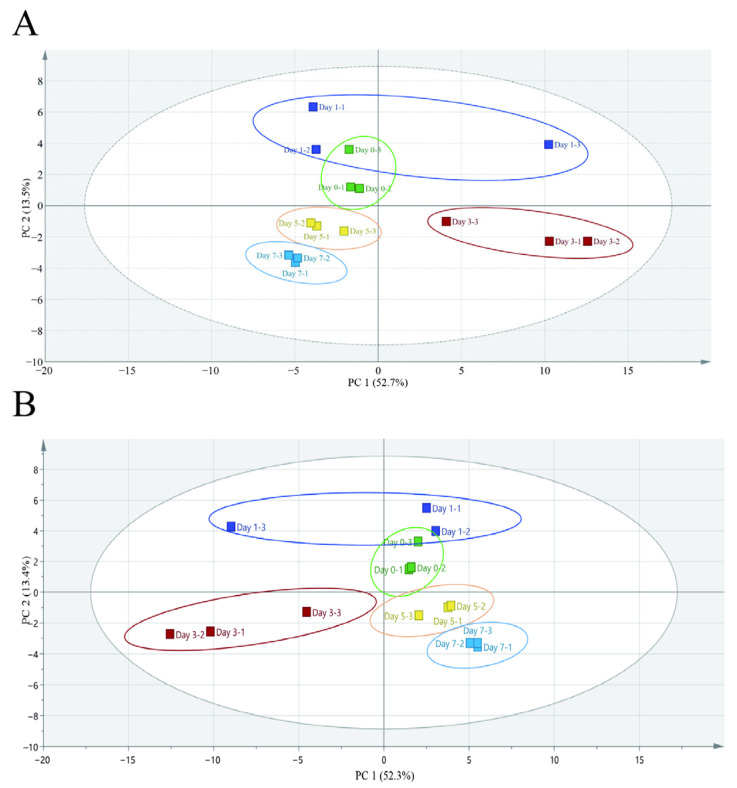
The PCA Score (**A**) and PLS-DA score (**B**) of the volatile profile of beef stored at 4 °C for one week (n = 3).

About 70 volatile metabolites were determined in the beef bottom round samples under refrigerated (4 °C) conditions. The volatile compounds were identified as aldehydes, ketones, alcohols, esters, hydrocarbons, eaters, acids, phenols, amines, amides, and other compounds. All volatile compounds were entered into the Simca software (version 14.1; Umetrics, Sweden) to perform unsupervised PCA. This analysis revealed group relationships and contributions to variance. Figure 4A shows the first and second principal components and explains 52.7 and 13.5% of the total variation, respectively, with a combined contribution of 66.2%. There was no overlap between days 1, 3, 5, and 7; however, there was an overlap between days 0 and 1. The results show that there were few changes in the volatile components of the beef samples at the initial storage time; however, the quality of the beef samples began to deteriorate from the third day. This explains the significantly distinct components in days 1 and 3. Although the PCA provides visual results, it does not elaborate on the overlaps; therefore, more analysis is needed.

The PCA score plot was further elaborated on using PLS-DA analysis. The first principal component explained the variation in 52.3% of cases, whereas the second principal component explained 13.4%. In 52.3% of situations, the first principal component described the variance, while in 13.4% of cases, the second principal component did so. The R2X and R2Y values were 0.903 and 0.964, respectively, which showed the model was stable. The Q2 value was 0.709, indicating a highly effective model prediction. As shown in Figure 4B, there was obvious clustering between different storage times.

#### 3.2.3. Analysis of Volatile Compounds for Quality Evaluation of Beef Bottom Round Samples Under Refrigeration Conditions (4 °C)

**Figure 5:**
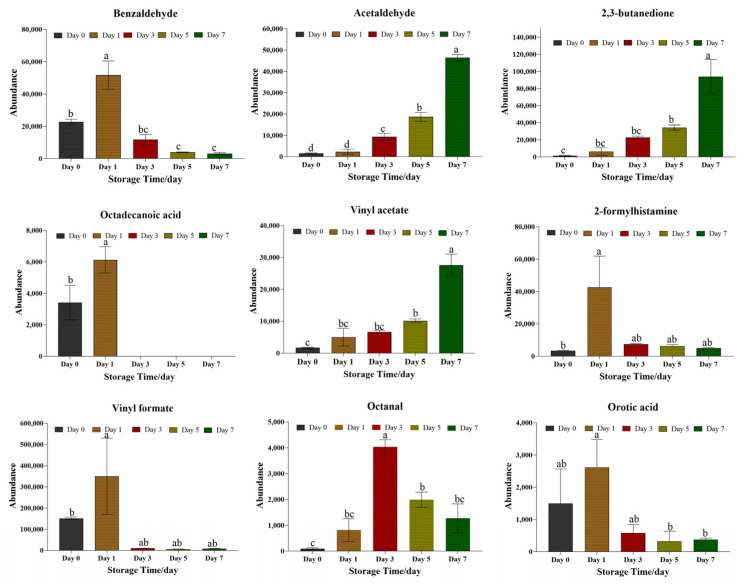
The abundance of volatile biomarkers in the beef bottom round samples under refrigeration conditions. The values are expressed as means ± SEM (n = 3). The a–d means represent significant differences (*p* < 0.05). Bars within the same storage time without the same letter (a, b, and c) were significantly different according to the Duncan post hoc test (*p* < 0.05).

Nine (9) volatile compounds were identified as potential biomarkers among multi-groups based on the VIP > 1 and *p* < 0.05 (see Table 2 and Figure 5). The compounds can be divided into five (5) distinct groups, namely: three aldehydes (benzaldehyde, acetaldehyde, and octanal); two esters (vinyl formate and vinyl acetate); two acids (octadecanoic acid and orotic acid); one ketone (2,3-butanedione); and one amine (2-formyl histamine). Storing beef samples under refrigerated conditions significantly increases their acetaldehyde, 2,3-butanedione, and vinyl acetate contents. Acetaldehyde exists in many species, it is an aldehydic, ethereal, and fruity-tasting compound. Previous studies have reported that acetaldehyde and 2,3-butanedione are associated with inoculated meat [50]. Acetaldehyde can be metabolized from alcohol, and can also be converted to acetic acid by the enzyme aldehyde dehydrogenase. The process involves glycolysis/gluconeogenesis, phenylalanine metabolism, benzoate degradation, taurine and hypotaurine metabolism, glycerophospholipid metabolism, microbial metabolism in diverse environments, and aromatic compound degradation. However, acetaldehyde is a known human carcinogen and might induce gastrointestinal tract carcinogenesis [51]. The other component (2,3-butanedione) provides a strong, sweet, buttery, and cheesy flavor profile. The resulting high concentration of 2,3-butanedione in the fresh beef samples could be attributed to microbial metabolism. Our results were consistent with Jing Bai’s findings, which reported that 2,3-butanedione generally increased with the spoilage grade [52]. In our study, the vinyl acetate concentration significantly increased on day 5, peaking at day 7. This is indicative of the fact that the beef samples must have been exposed to pathogenic microorganism contamination.

The concentration of benzaldehyde, octadecanoic acid, vinyl formate, 2-formyl histamine, and orotic acid all peaked at the initial measurement (day 1) and declined thereafter. Benzaldehyde has an strong, sharp, sweet, and bitter almond, burnt sugar, and cherry flavor, and is mainly involved in toluene degradation, aminobenzoate degradation, microbial metabolism, and the degradation of aromatic compounds. The change in benzaldehyde concentration could be due to lipid oxidation which can induce protein oxidation to form dicarbonyl compounds [53]. The large amounts of aldehydes can result in an unpleasant odor. Our results show that the beef samples were in the process of becoming putrefied. The change in octadecanoic acid was consistent with benzaldehyde; this was because the octadecanoic acid, as a lipid, could be metabolized to aldehydes. Octadecanoic acid is a source of aroma. The 2-formyl histamine compound is a common volatile organic associated with cold food storage and results from the microbial breakdown of lipids and the hydrolysis of amino acids. Orotic acid provides a sour and fruity flavor and is involved in pyrimidine metabolism and the biosynthesis of cofactors. It is also found in milk and commonly exists in the synthetic pathway of pyrimidine nucleotides of various microbial variants [54]. Its presence in beef samples is indicative of beef putrefaction. Octanal had a slow and gradual incline, peaking on the third day, with a sharp decline afterward. It has an aldehydic, citrus, and fat-tasting aroma and might have resulted from lipid oxidation. In Li Gang Yu’s study, octanal was regarded as an indicator of quality deterioration of beef during frozen storage [55].

## 4. Conclusions

The present study reveals the quality dynamics of the beef bottom round both during 2-month frozen storage (−18 °C) and week-long refrigerated storage (4 °C). During the long-term frozen storage and the short-term refrigerated storage, an unsatisfactory surface color and pH could directly affect the consumers’ purchasing desire. The cooking loss, thawing loss, centrifugal loss, drip loss, moisture content, shear force, TPA, and TBARS data together indicate that long-term frozen storage can significantly reduce the WHC and affect the texture and results of lipid oxidation. The VOCs identified during the short-term refrigerated storage illustrate that the flavor undergoes significant changes with prolonged storage. In brief, both long-term frozen storage and short-term refrigerated storage can lead to significant quality changes in the beef bottom round. The findings of this study will provide novel insights for the development of innovative beef preservation methods.

## Figures and Tables

**Table 1:** Texture profile analysis of beef bottom round stored under −18 °C (n = 5, mean ± SEM).

Specification	Unit of Measure	Storage Time		
		Day 0	Day 30	Day 60
Hardness	N	328.21 ^a^ ± 33.61	163.11 ^b^ ± 21.05	74.70 ^c^ ± 7.53
Adhesiveness	g/s	212.27 ^a^ ± 24.96	144.89 ^b^ ± 4.76	59.36 ^c^ ± 4.60
Springiness	-	0.86 ^a^ ± 0.03	0.44 ^b^ ± 0.02	0.36 ^c^ ± 0.01
Cohesiveness	-	0.43 ^b^ ± 0.04	0.47 ^ab^ ± 0.02	0.55 ^a^ ± 0.01
Gumminess	N	156.21 ^a^ ± 12.19	82.50 ^b^ ± 7.8	51.99 ^c^ ± 6.54
Chewiness	N	58.52 ^a^ ± 5.45	30.60 ^b^ ± 4.81	22.41 ^b^ ± 2.16
Resilience	-	0.29 ± 0.02	0.29 ± 0.05	0.31 ± 0.03

Data represent the mean ± SEM of triplicate experiments. Different lowercase letters in identical columns indicate a significant difference (*p* < 0.05).

**Table 2:** The biomarkers identified in the beef bottom round samples during the refrigerated storage period.

Count	Compound	Formula	Flavors	Matched Pathways
1	Benzaldehyde	C_7_H_6_O	Almond, burnt sugar, cherry, strong, sharp, sweet, bitter	Degradation of phenylalanine, Aminobenzoate degradation, Microbial metabolism in diverse environments, Degradation of aromatic compounds
2	2,3-butanedione	C_4_H_6_O_2_	Sweet, buttery	Butanoate metabolism
3	Acetaldehyde	C_2_H_4_O	Pungent, ether, whiskey, ethereal, aldehydic, fruity	Glycolysis/Gluconeogenesis, Phenylalanine metabolism, Benzoate degradation, Taurine and hypotaurine metabolism, Glycerophospholipid metabolism, Microbial metabolism in diverse environments, Degradation of aromatic compounds
4	Octadecanoic acid	C_18_H_36_O_2_	Rich, smooth	Fatty acid biosynthesis Biosynthesis of unsaturated fatty acids Biosynthesis of plant secondary metabolites
5	Vinyl formate	C_3_H_4_O_2_	NA	NA
6	2-formylhistamine	C_6_H_9_N_3_O	Cabbage, garlic, amine, urine	NA
7	Vinyl acetate	C_4_H_6_O_2_	NA	NA
8	Octanal	C_8_H_16_O	Aldehydic, citrus, fat	NA
9	Orotic acid	C_5_H_4_N_2_O_4_	Sour, fruity	Pyrimidine metabolism, Biosynthesis of cofactors

## Data Availability

The original contributions presented in the study are included in the article; further inquiries can be directed to the corresponding author.
